# Proposal of an Algorithm for the Clinical and Molecular Diagnosis of RASopathies Based on HPO Nomenclature

**DOI:** 10.3390/ijms27167348

**Published:** 2026-08-17

**Authors:** Fernanda Meneses, Carlos Quintero, Juliana Lores, Eidith Gómez-Pineda, Diana Ramírez-Montaño, Estephania Candelo, Harry Pachajoa

**Affiliations:** 1 Facultad de Ciencias de la Salud, Universidad Icesi, Cali 760032, Colombia; fernandameneses09@gmail.com (F.M.); carlosqr478@gmail.com (C.Q.); eidith1721@gmail.com (E.G.-P.); 2 Departamento de Ciencias Básicas, Facultad de Ciencias de la Salud, Pontificia Universidad Javeriana, Cali 760032, Colombia; julianalores4@gmail.com; 3 Unidad de Medicina Genómica y Genética, Clínica Imbanaco Grupo Quironsalud, Cali 760032, Colombia; diana.ramirezm@quironsalud.com; 4 Hospital Universitario del Valle, Cali 76003, Colombia; 5 Departamento de Morfología, Facultad de Ciencias de la Salud, Universidad del Valle, Cali 760032, Colombia; 6 Department of Otolaryngology and Head-Neck Surgery, University of Washington, Seattle, WA 98195, USA; estephaniacandelo12@gmail.com; 7 Servicio de Genética Médica, Fundación Valle del Lili, Cali 760032, Colombia; 8 Centro de Investigación en Anomalías Congénitas y Enfermedades Raras (CIACER), Universidad Icesi, Cali 760031, Colombia; 9 Centro de Investigaciones Clínicas, Fundación Valle del Lili, Cali 760032, Colombia

**Keywords:** RASopathies, Human Phenotype Ontology, diagnostic algorithm, rare genetic disorder

## Abstract

RASopathies are a group of genetic disorders caused by germline variants affecting the RAS/MAPK pathway. Their shared phenotypic features—craniofacial anomalies, cardiac defects, cutaneous findings, neurodevelopmental issues, and cancer predisposition—make diagnosis challenging, especially since most lack standardized clinical criteria. This study aimed to develop a practical diagnostic algorithm based on high-frequency Human Phenotype Ontology (HPO) features. Key clinical variables for each RASopathy were identified through HPO, PubMed, and GeneReviews. Only findings present in 80–99% of cases or supported by expert consensus were included. A decision-tree algorithm was constructed and preliminarily evaluated using a blinded cohort of 50 individuals with confirmed molecular diagnoses. Patients were eligible for inclusion if they met the following criteria: (1) molecularly confirmed diagnosis of a RASopathy by next-generation sequencing identifying a pathogenic or likely pathogenic variant; (2) availability of complete phenotypic records in the institutional clinical database; and (3) age at evaluation between 0 and 18 years. Patients were excluded if phenotypic data were incomplete or if molecular confirmation was absent. The algorithm integrates phenotypic patterns and genotype–phenotype correlations. Validation showed 78% accuracy (95% CI: 64.0–88.4%) for clinical diagnosis and 66% accuracy (95% CI: 51.2–78.8%) for molecular prediction. To our knowledge, this is the first HPO-based diagnostic algorithm for the clinical and molecular approach to RASopathies. It provides a structured, accessible tool to improve early recognition and guide molecular testing, particularly for the RASopathy subtypes represented in the validation cohort. Further external validation including underrepresented subtypes is required.

## 1. Introduction

RASopathies are a group of genetic disorders caused by pathogenic germline variants in the genes encoding regulatory elements of the RAS/mitogen-activated protein kinase (MAPK) pathway. This molecular pathway is an intracellular signaling cascade involved in the regulation of the cellular cycle, proliferation, differentiation and cellular death [1]. Together, they are one of the largest known groups of malformation syndromes, affecting approximately 1 in 1000 people [2]. Each RASopathy expresses a characteristic phenotypic pattern; however, the common mechanisms of dysregulation of the RAS/MAPK pathway cause overlapping clinical manifestations such as craniofacial dysmorphology, cardiac malformations, skin abnormalities, neurocognitive impairment and an increased risk of cancer [3], which makes early diagnosis difficult.

Currently, there are criteria for the clinical diagnosis of Neurofibromatosis type 1 [4,5,6] and Noonan Syndrome [7,8], while other RASopathies lack a clinical standard for their diagnosis. The foregoing, added to the difficult access to a molecular diagnosis, might be contributing to these pathologies being underdiagnosed entities.

The objective of this study is to propose an algorithm to approach the clinical and molecular diagnosis of patients with suspected RASopathies based on the analysis of high-frequency HPO-defined phenotypic features [9].

## 2. Results

### 2.1. Algorithm Construction

**Figure 1:**
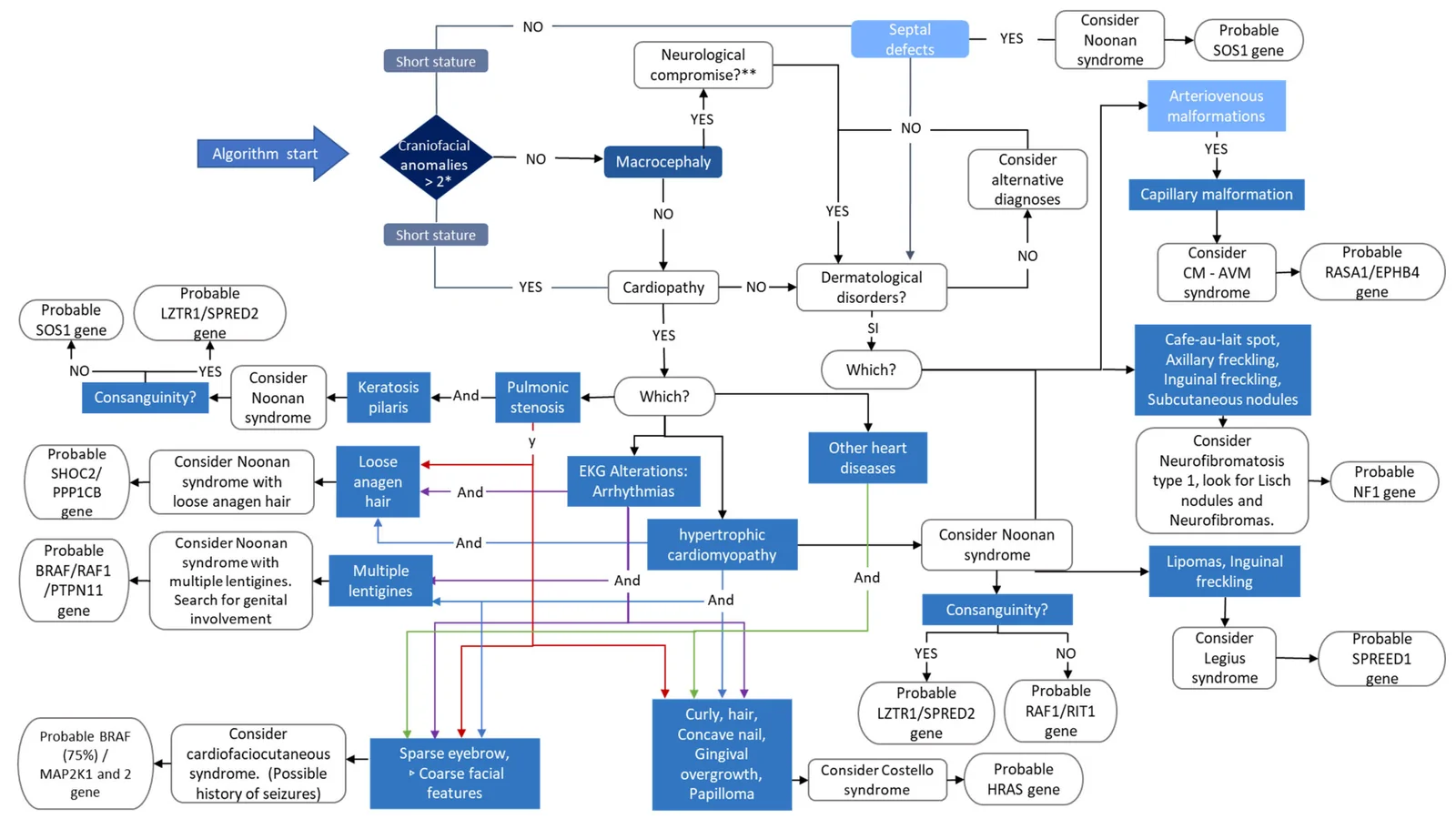
Algorithm for the diagnostic approach of RASopathies. ◇ Algorithm start. * Short neck, pulled neck, broad forehead, hypertelorism, posteriorly rotated low-set ears, downward slanting palpebral fissures and depressed nasal bridge. ** Intellectual disability, delayed psychomotor development and delayed speech. ▹ Coarse facial features, defined by the presence of long face, anteverted nostrils and widened ear helix. EKG: Electrocardiogram, MC-MAV: Capillary malformation–arteriovenous malformation syndrome, SD: syndrome.

According to the high frequency of presentation in RASopathies, a set of clinical patterns for each RASopathy were obtained. Genotype–phenotype correlation was also accounted for (Table 1), and both patterns were integrated in a diagnostic algorithm able to suggest both a clinical and molecular diagnosis for each case (Figure 1).

### 2.2. Algorithm Validation

**Figure 2:**
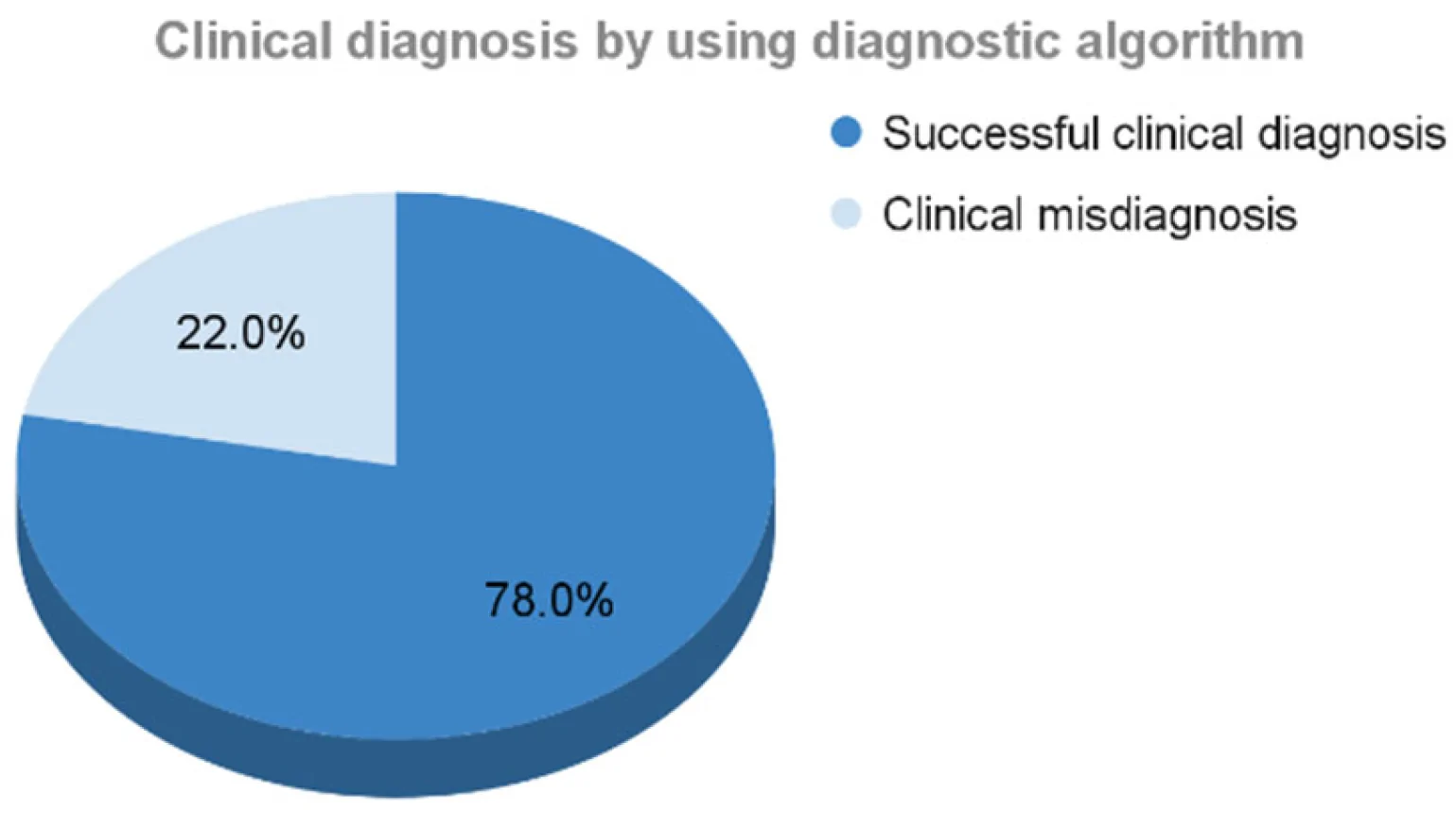
Clinical diagnosis accuracy of the HPO-based RASopathy diagnostic algorithm. Bar chart shows the proportion of correctly and incorrectly classified patients (*n* = 50) based on clinical syndrome prediction by the algorithm, compared against molecularly confirmed diagnoses as the reference standard. Of 50 patients, 39 (78%) received a correct clinical syndrome prediction (accuracy: 78%; 95% CI: 64.0–88.4%, calculated using the Wilson score method). Correct classification was defined as concordance between the algorithm-predicted syndrome and the molecularly confirmed diagnosis. Patients classified as ‘Consider other diagnosis’ by the algorithm were counted as incorrect predictions.

**Figure 3:**
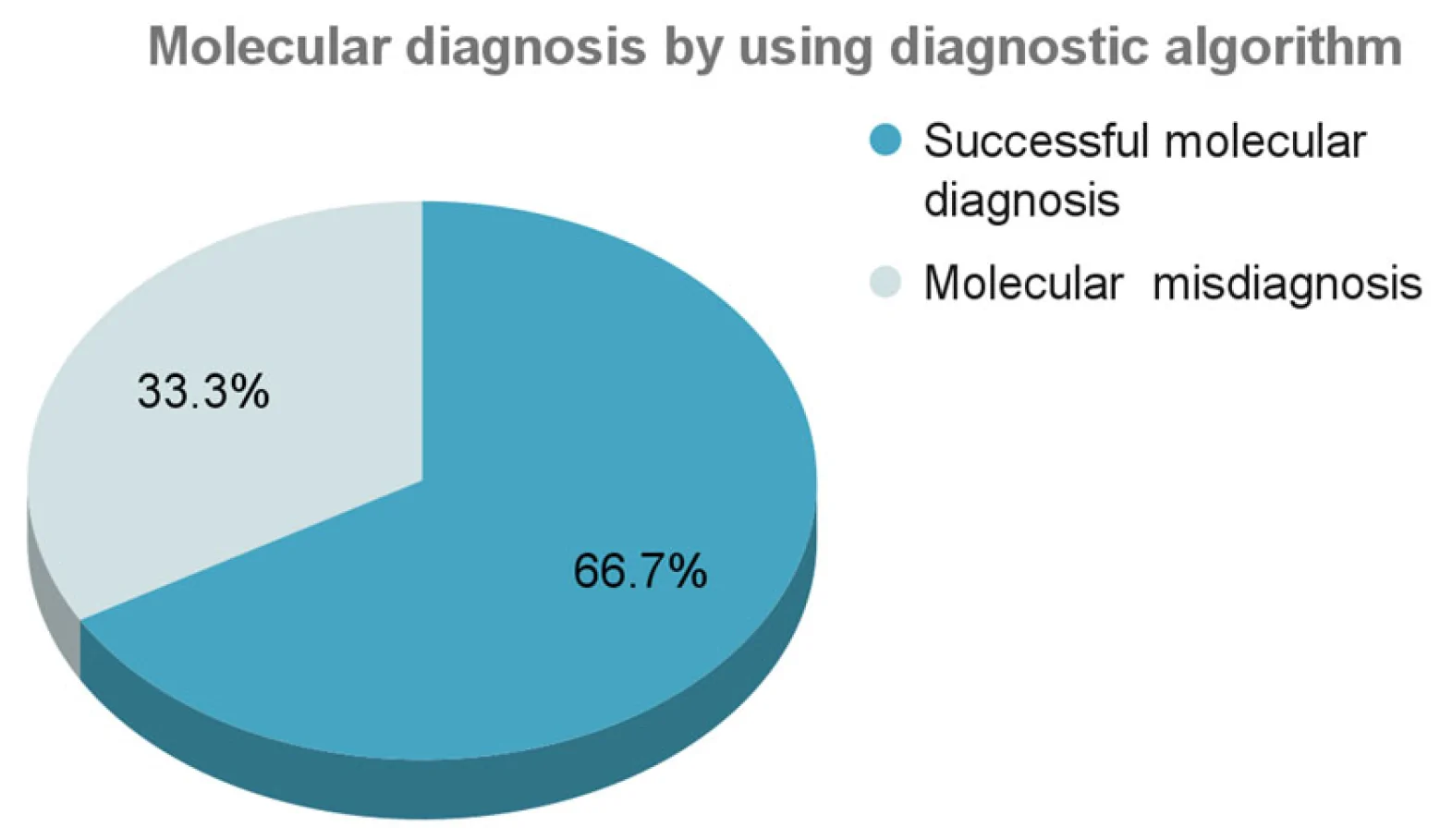
Molecular diagnosis accuracy of the HPO-based RASopathy diagnostic algorithm. Bar chart showing the proportion of correctly and incorrectly predicted causative genes (*n* = 50) based on molecular gene prediction by the algorithm, compared against the confirmed pathogenic or likely pathogenic variant identified by next-generation sequencing. Of 50 patients, 33 (66%) received a correct molecular gene prediction (accuracy: 66%; 95% CI: 51.2–78.8%, calculated using the Wilson score method). The lower molecular accuracy relative to clinical accuracy (78%) reflects within-syndrome molecular heterogeneity and multiple genes (e.g., PTPN11, SOS1, RAF1, KRAS, LZTR1) can underlie Noonan syndrome, making gene-level prediction inherently more challenging than syndrome-level prediction. Cases where the algorithm correctly identified the clinical syndrome but predicted an incorrect causative gene are captured here as molecular misclassifications.

We compared the suggested diagnosis proposed by the algorithm to the confirmed molecular diagnosis of a database of 50 RASopathy patients. Table 2 summarizes the diagnosis distribution in each individual. We were able to obtain a 78% positive rate of accurate clinical diagnosis (Figure 2); on the other hand, there was a 66% rate of accurate molecular diagnosis (Figure 3).

Supplementary Table S1 provides the full patient-level dataset including age, sex, molecular diagnosis, causative gene, algorithm-predicted diagnosis, and reason for misclassification for all 50 patients. Supplementary Table S2 presents the inter-rater agreement data (Cohen’s kappa κ = 0.82; 95% CI: 0.71–0.93). Supplementary Table S3 provides the multiclass confusion matrix for algorithm predictions at the syndrome level. Supplementary Table S4 lists all GeneReviews sources used for phenotype frequency extraction. The full RASopathy diagnostic algorithm and the step-by-step clinical use pathway are provided as Supplementary Figures S1 and S2, respectively.

## 3. Discussion

We developed an algorithm to guide clinical and molecular diagnosis for the different syndromes included within the RASopathies, aimed at specialized and non-specialized health personnel, based solely on high-frequency phenotypic findings (80–99%) indexed in the HPO virtual library. A first validation was carried out in a cohort of 50 affected individuals, with effectiveness in clinical diagnosis in 78% of cases and molecular diagnosis in 66% of cases.

To illustrate the practical application of the algorithm in routine clinical settings, Figure S1 in the Supplementary Material presents a step-by-step clinical use pathway. In this workflow, the general practitioner or paediatrician performs an initial clinical examination and applies the algorithm to the patient’s phenotypic features. A high-probability syndrome identification prompts referral to a clinical geneticist, who confirms the diagnosis through molecular testing. Confirmed cases then receive genetic counselling and syndrome-specific clinical management. This pathway demonstrates how the algorithm can function as a triage tool, reducing diagnostic delay particularly in settings with limited access to specialist genetics services. This workflow is illustrated in Figure S2 of the Supplementary Material.

The perception of experts in RASopathies is that there is a general lack of knowledge about these syndromes, even among health specialists. This fact entails, as in many other rare diseases, a significant delay in diagnosis, which makes it difficult to establish corrective actions in the first years of life, a critical period for preventing permanent damage to multiple systems, which is why there is a need for accessible diagnostic tools applicable across different levels of healthcare.

A search was carried out in PubMed using the search terms “algorithm”, “RASopathies” OR “RASopathy” with which four results were obtained; however, no publication was aimed at suggesting a diagnostic approach. Therefore, to date, there are no published studies that seek to establish a methodical and standardized approach for the clinical and molecular diagnosis of RASopathies.

For a few decades, algorithms have gained a relevant role in medicine, allowing a methodical and systematic approach to a clinical problem, whether diagnostic or therapeutic. Limited access to molecular testing may contribute to underdiagnosis of these conditions in low-income settings; thus, these clinical models function as a guide for health personnel in general, achieving standardization of clinical results and minimization of error in clinical practice. Similar studies have been published in recent years, seeking to propose a diagnostic algorithm for groups of heterogeneous genetic diseases aimed at non-specialized health personnel. It is worth highlighting the publication of Araújo et al. [10] in which a diagnostic approach is proposed for lipodystrophies and the first validation reaches diagnostic certainty in around 80% of cases.

Applying this formula to the validation cohort (*n* = 50), clinical syndrome prediction yielded 39 correctly classified patients (accuracy 78%; 95% CI: 64.0–88.4%); molecular gene prediction yielded 33 correctly classified patients (accuracy 66%; 95% CI: 51.2–78.8%). Because the validation cohort consisted exclusively of molecularly confirmed RASopathy cases, no true-negative individuals were available; consequently, specificity and negative predictive value cannot be reliably estimated from this dataset.

It is important to note that ClinVar and OMIM, while invaluable resources for variant interpretation and gene–disease associations, were not used as cross-validation benchmarks in this study. This decision reflects a fundamental methodological distinction: ClinVar and OMIM are genotype-centric databases designed to support variant classification and disease–gene mapping, not to validate phenotype-based clinical screening algorithms. Our algorithm was developed entirely from phenotypic information derived from HPO, GeneReviews, and peer-reviewed literature, and was validated against molecularly confirmed diagnoses as the gold standard. Using genotype-centric databases to evaluate a phenotype-driven triage tool would introduce a category mismatch and would not constitute a valid form of algorithmic performance assessment. Phenotypic-to-genotypic validation—as performed here—remains the appropriate methodological standard for this type of diagnostic aid.

The limitations of our research include the absence of frequency data reported in HPO. This reflects the epidemiology of rare diseases, where patient recruitment is limited by low prevalence, geographic dispersion, and restricted availability of molecularly confirmed cases [11,12]. Future versions of the algorithm could incorporate weighted low-frequency features or syndrome-specific variables to improve diagnostic specificity without compromising its applicability as an initial screening tool. Future multicenter studies including larger and more balanced cohorts across all RASopathy subtypes will be necessary to further evaluate its generalizability and clinical utility for some phenotypic findings, which were included according to the opinion of expert geneticists in the diagnosis of RASopathies. Further limitations include the small validation cohort of only 50 patients, and few different diagnoses, including neurofibromatosis type I, Noonan syndrome, and Noonan syndrome-like with loose anagen hair. Therefore, the algorithm’s applicability for the diagnosis of capillary malformation–arteriovenous malformation syndrome, Costello syndrome, Noonan syndrome with multiple lentigines, Legius syndrome and Cardiofaciocutaneous syndrome was not validated. Further validation with a larger number of patients and diagnoses is required. The possibility of optimizing the diagnostic algorithm remains open as new technologies develop and new evidence is published.

These limitations are consistent with the inherent challenges of research in rare diseases, where patient recruitment is constrained by low prevalence, geographic dispersion, and restricted availability of molecularly confirmed cases [11,12]. Future versions of the algorithm could incorporate weighted low-frequency clinical features or syndrome-specific variables to improve diagnostic specificity without compromising its value as an initial primary-care screening tool. Future multicenter studies including larger and more balanced cohorts across all RASopathy subtypes will be necessary to evaluate generalizability and support broader clinical implementation.

## 4. Materials and Methods

### 4.1. Algorithm Construction

**Figure 4:**
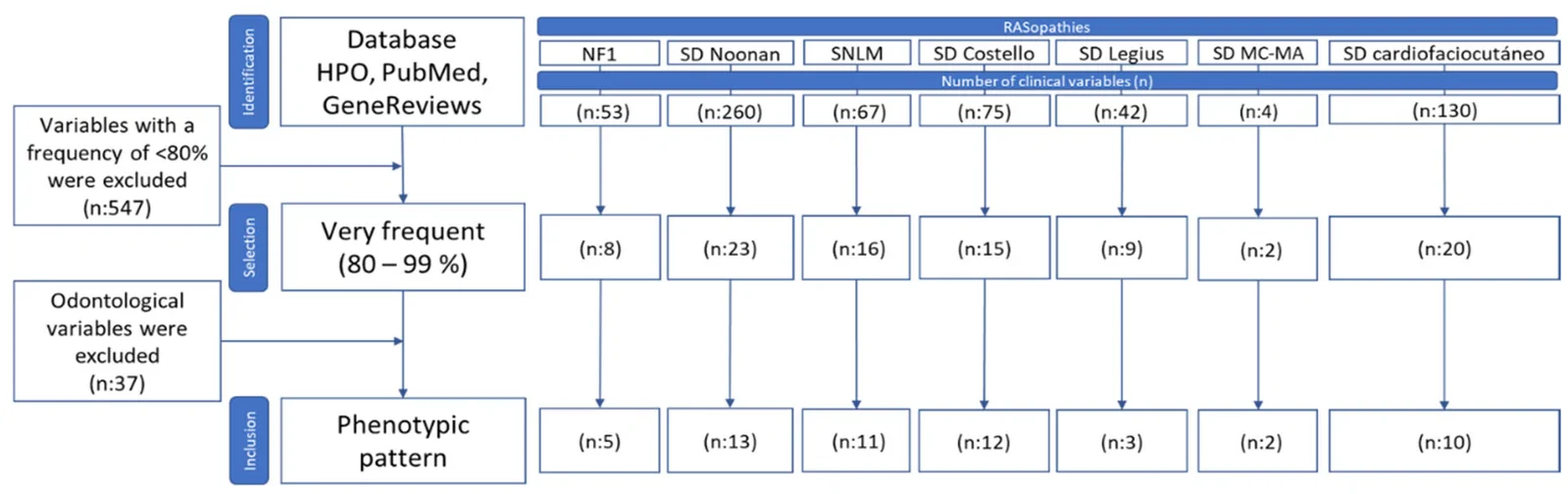
Selection of clinical variables. MC-MA: Capillary malformation–arteriovenous malformation, SD: syndrome, SNLM: Noonan syndrome with multiple lentigines.

Initially, a search was made in the HPO virtual library and in the literature (PubMed, GeneReviews) for each RASopathy, with the aim of identifying different clinical variables that could guide each diagnosis of RASopathy. For each gene-associated syndrome, a systematic phenotype extraction was performed using three complementary sources: (1) Human Phenotype Ontology (HPO) database (accessed January 2024), (2) GeneReviews—specifically the “Frequency of Select Features” sections for Noonan Syndrome (NBK1124), Cardiofaciocutaneous Syndrome (NBK1186), Legius Syndrome (NBK47312), Noonan Syndrome with Multiple Lentigines (NBK1383), Costello Syndrome (NBK1507), and Neurofibromatosis type 1 (NBK1109)—and (3) PubMed, searched using predefined MeSH term combinations: “Noonan Syndrome” [Mesh] AND “Phenotype” [Mesh]; “Cardiofaciocutaneous syndrome” [Supplementary Concept] AND “Phenotype” [Mesh]; “Legius syndrome” [Supplementary Concept] AND “Phenotype” [Mesh]; “LEOPARD Syndrome” [Mesh] AND “Phenotype” [Mesh]; “Costello Syndrome” [Mesh] AND “Phenotype” [Mesh]; “Neurofibromatosis 1” [Mesh] AND “Phenotype” [Mesh]. References were manually reviewed for inclusion. Dermatological, cardiac and craniofacial variables were considered. Four genes associated with RASopathy according to HPO were excluded because of lack of evidence after a review of the literature and the opinion of a geneticist with experience in diagnosing RASopathies (*A2ML1*, *RASA2*, *SPRY1*, *MYST4*). Subsequently, the frequency of these variables in each RASopathy was examined, considering the high-frequency clinical characteristics to be those reported in the literature (HPO, GeneReviews, PubMed) as occurring in approximately 80–99% of affected individuals with a given RASopathy, which was not the frequency observed in our validation cohort of 50 patients; therefore, the variables that appear in a lower percentage were discarded. Because the objective of this study was to develop a screening tool for primary care, this threshold was defined a priori to prioritize highly prevalent features, maximizing the sensitivity and clinical applicability of the proposed algorithm during the initial evaluation. Lower-frequency manifestations were excluded from the screening model, although they may be useful during specialist assessment. In addition, some clinical characteristics did not show a frequency value by HPO; thus, they were analyzed based on a review of many descriptive studies published with multiple patients, and they were also discussed with a clinical geneticist with experience in diagnosing RASopathies to define inclusion or not in the study. Dental variables were excluded due to the difficulty of identifying them in the basic clinical context of the general physician (Figure 4).

With the collected information, the algorithm was designed as a decision tree based on dichotomous questions (“Yes/No”), and direct open questions (“Which one?”) based on the high-frequency phenotypic patterns for each of the RASopathies, and in some cases, if there was evidence of enough genotype-phenotype correlation for each gene within a syndrome (Figure 1).

Considering the organization of the algorithm based on the clinical examination in an average medical setting, the craniofacial anomalies will be first identified, followed by the presence or absence of short stature. For the neurological, dermatological and cardiac manifestations, colored lines have been added to help follow the algorithm’s decisions.

For example, a patient with a short neck, hypertelorism, sparse eyebrows and short stature, in addition to pulmonary stenosis, will be categorized as cardiofaciocutaneous syndrome, probably associated with genes such as *BRAF*, *MAP2K1* or *MAP2K2*.

### 4.2. Algorithm Validation

Clinical data from a cohort of individuals with confirmed molecular diagnosis of different RASopathies were used as part of the ongoing study “Clinical and molecular characterization of patients with RASopathies in Colombia” in Fundación Valle del Lili University Hospital [13].

The information indexed in this database was provided to two researchers who applied the algorithm to each of the 50 individuals based on their phenotypic characteristics. Both researchers applied the algorithm independently and in a blinded manner. Discrepancies between raters were identified and resolved by consensus between the two researchers. Inter-rater agreement was assessed using Cohen’s kappa coefficient (κ = 0.82, 95% CI: 0.71–0.93), indicating strong agreement. It is important to mention that during this process, the data were blinded and only an ID number was given. Subsequently, a comparison was made considering the diagnosis suggested by the algorithm and the confirmed molecular diagnosis found in the database. Thus, with the above information, the concordance proportion of these diagnoses was calculated.

Supplementary Table S1 provides the full patient-level dataset including age, sex, molecular diagnosis, causative gene, algorithm-predicted diagnosis, and reason for misclassification for all 50 patients. Supplementary Table S2 presents the inter-rater agreement data (Cohen’s kappa κ = 0.82; 95% CI: 0.71–0.93). Supplementary Table S3 provides the multiclass confusion matrix for algorithm predictions. Supplementary Table S4 lists all GeneReviews sources used for phenotype frequency extraction. The RASopathy Diagnostic Algorithm and the Clinical Use Pathway are provided as Figures S1 and S2, respectively, in the Supplementary Material.

For the statistical analysis, the performance of the proposed algorithm was evaluated by comparing the algorithmically predicted diagnosis with the molecular diagnosis, which served as the reference standard. Molecular diagnosis was established by the identification of a pathogenic or likely pathogenic variant in a RASopathy-associated gene by means of next-generation sequencing. Accuracy was calculated using the following formula: Accuracy (%) = (Number of correctly classified patients/Total number of evaluated patients) × 100. A case was considered correctly classified when the syndrome predicted by the algorithm was concordant with the molecular diagnosis. Because our validation cohort consisted exclusively of molecularly confirmed RASopathy cases, no true-negative individuals were available; consequently, specificity and negative predictive values cannot be estimated. The 95% confidence intervals for accuracy were calculated using the Wilson score method.

The accuracy formula applied was as follows: Accuracy (%) = (Number of correctly classified patients/Total number of evaluated patients) × 100, where z = 1.96 for 95% confidence. A case was considered correctly classified when the syndrome predicted by the algorithm was concordant with the molecularly confirmed diagnosis. The 95% confidence intervals (CIs) were calculated using the Wilson score method: CI = [$\hat{p}$ + z^2^/(2n) ± z√($\hat{p}$(1 − $\hat{p}$)/n + z^2^/(4n^2^))]/(1 + z^2^/n), where $\hat{p}$ is the observed accuracy and n is the total number of patients evaluated.

## Figures and Tables

**Table 1:** Summary of the phenotypical pattern of each RASopathy.

RASopathies	Clinical Features According to HPO Nomenclature	Genes	Genotype–Phenotype Correlation (>80%)
Neurofibromatosis type 1	Iris hamartomas (HP:0009737) Multiple cafe-au-lait spots (HP:0007565) Neurofibromas (HP:0001067) Plexiform neurofibroma (HP:0009732) Subcutaneous nodule (HP:0001482) Axillary freckling (HP:0000997) Inguinal freckling (HP:0030052)	*NF1*	-
Capillary malformation–arteriovenous malformation syndrome	Multifocal capillary malformations * Arteriovenous malformation (HP:0100026)	*RASA1*	-
		*EPHB4*	-
Noonan syndrome with multiple lentigines	Short neck (HP:0000470) Broad forehead (HP:0000337) Hypertelorism (HP:0000316) Low-set ears (HP:0000369) Posteriorly rotated ears (HP:0000358) Downslanted palpebral fissures (HP:0000494) Depressed nasal bridge (HP:0005280) Short stature (HP:0004322) Multiple lentigines (HP:0001003) Abnormal EKG (HP:0003115) Hypertrophic cardiomyopathy (HP:0001639)	*BRAF*	-
		*RAF1*	-
		*PTPN11*	-
Noonan syndrome	Short neck (HP:0000470) Broad forehead (HP:0000337) Hypertelorism (HP:0000316) Low-set ears (HP:0000369) Posteriorly rotated ears (HP:0000358) Downslanted palpebral fissures (HP:0000494) Depressed nasal bridge (HP:0005280) Short stature (HP:0004322)	*SHOC2/PPP1CB* *(Gene reviews)*	Loose anagen hair (HP:0040169) Sparse scalp hair (HP:0002209)
		*BRAF*	-
		*PTPN11*	-
		*MAP2K1*	-
		*SOS1*	Keratosis pilaris (HP:0032152) Abnormal cardiac septum morphology (HP:0001671) Short stature (HP:0004322)
		*KRAS*	-
		*RAF1*	Hypertrophic cardiomyopathy (HP:0001639)
		*SOS2*	-
		*NRAS*	-
		*LZTR1*	-
		*MRAS*	-
		*RRAS2*	-
		*CBL*	-
		*SPRED2*	-
		*RIT1*	Hypertrophic cardiomyopathy (HP:0001639)
Costello syndrome	Short neck (HP:0000470) Broad forehead (HP:0000337) Hypertelorism (HP:0000316) Low-set ears (HP:0000369) Posteriorly rotated ears (HP:0000358) Downslanted palpebral fissures (HP:0000494) Depressed nasal bridge (HP:0005280) Short stature (HP:0004322) Curly hair (HP:0002212) Concave nail (HP:0001598) Papilloma (HP:0012740) Gingival overgrowth (HP:0000212)	*HRAS*	-
Cardiofaciocutaneous syndrome	Short neck (HP:0000470) Broad forehead (HP:0000337) Hypertelorism (HP:0000316) Low-set ears (HP:0000369) Posteriorly rotated ears (HP:0000358) Downslanted palpebral fissures (HP:0000494) Depressed nasal bridge (HP:0005280) Short stature (HP:0004322) Coarse facial features (HP:0000280) Aplasia/Hypoplasia of the eyebrow (HP:0100840)	*BRAF*	-
		*MAP2K1*	-
		*MAP2K2*	-
		*KRAS*	-
Legius syndrome	Multiple cafe-au-lait spots (HP:0007565) Axillary freckling (HP:0000997) Inguinal freckling (HP:0030052)	*SPRED1*	-

* Clinical characteristics not indexed in Human Phenotype Ontology. However, they were included in the study, by consideration of specialists in clinical genetics.

**Table 2:** Summary of the diagnosis distribution in each individual.

Patient	Algorithm-Suggested Diagnosis (3)	Algorithm-Suggested Gene	Confirmed Clinical and Molecular Diagnosis (2)
**1**	Noonan syndrome	*PTPN11*	Noonan syndrome—*PTPN11*
**2**	Consider other diagnosis	*-*	Noonan syndrome—*PTPN11*
**3**	Noonan syndrome	*PTPN11*	Noonan syndrome—*PTPN11*
**4**	Neurofibromatosis type I	*NF1*	Noonan syndrome—*PTPN11*
**5**	Noonan syndrome	*PTPN11*	Noonan syndrome—*PTPN11*
**6**	Noonan syndrome	*PTPN11*	Noonan syndrome—*SOS1*
**7**	Noonan syndrome	*PTPN11*	Noonan syndrome—*PTPN11*
**8**	Noonan syndrome	*PTPN11*	Noonan syndrome—*KRAS*
**9**	Noonan syndrome with multiple lentigines	*BRAF/RAF1/PTPN11*	Noonan syndrome—*RAF1*
**10**	Noonan syndrome	*PTPN11*	Noonan syndrome—*PTPN11*
**11**	Neurofibromatosis type I	*NF1*	Noonan syndrome—*BRAF*
**12**	Noonan syndrome	*PTPN11*	Noonan syndrome—*PTPN11*
**13**	Noonan syndrome	*PTPN11*	Noonan syndrome—*RAF1*
**14**	Noonan syndrome	*PTPN11*	Noonan syndrome—*PTPN11*
**15**	Consider other diagnosis	*-*	Noonan syndrome—*PTPN11*
**16**	Noonan syndrome	*PTPN11*	Noonan syndrome—*SOS1*
**17**	Noonan syndrome	*PTPN11*	Noonan syndrome—*PTPN11*
**18**	Noonan syndrome	*PTPN11*	Noonan syndrome—*PTPN11*
**19**	Consider other diagnosis	*PTPN11*	Noonan syndrome—*PTPN11*
**20**	Noonan syndrome	*PTPN11*	Noonan syndrome-like with loose anagen hair—*SHOC2*
**21**	Neurofibromatosis type I	*NF1*	Noonan syndrome-like with loose anagen hair—*SHOC2*
**22**	Costello syndrome	*HRAS*	Noonan syndrome-like with loose anagen hair—*SHOC2*
**23**	Noonan syndrome	*PTPN11*	Noonan syndrome—*PTPN11*
**24**	Noonan syndrome	*PTPN11*	Noonan syndrome—*PTPN11*
**25**	Noonan syndrome	*PTPN11*	Noonan syndrome—*SOS1*
**26**	Consider other diagnosis	*-*	Noonan syndrome—*PTPN11*
**27**	Noonan syndrome	*LZTR1*	Noonan syndrome—*LZTR1*
**28**	Consider other diagnosis	-	Neurofibromatosis type I—*NF1*
**29**	Neurofibromatosis type I	*NF1*	Neurofibromatosis type I—*NF1*
**30**	Neurofibromatosis type I	*NF1*	Neurofibromatosis type I—*NF1*
**31**	Neurofibromatosis type I	*NF1*	Neurofibromatosis type I—*NF1*
**32**	Neurofibromatosis type I	*NF1*	Neurofibromatosis type I—*NF1*
**33**	Neurofibromatosis type I	*NF1*	Neurofibromatosis type I—*NF1*
**34**	Neurofibromatosis type I	*NF1*	Neurofibromatosis type I—*NF1*
**35**	Neurofibromatosis type I	*NF1*	Neurofibromatosis type I—*NF1*
**36**	Neurofibromatosis type I	*NF1*	Neurofibromatosis type I—*NF1*
**37**	Neurofibromatosis type I	*NF1*	Neurofibromatosis type I—*NF1*
**38**	Neurofibromatosis type I	*NF1*	Neurofibromatosis type I—*NF1*
**39**	Neurofibromatosis type I	*NF1*	Neurofibromatosis type I—*NF1*
**40**	Consider other diagnosis	*-*	Noonan syndrome—*PTPN11*
**41**	Noonan syndrome	*SOS1*	Noonan syndrome—*PTPN11*
**42**	Noonan syndrome	*PTPN11*	Noonan syndrome—*PTPN11*
**43**	Noonan syndrome	*PTPN11*	Noonan syndrome—*PTPN11*
**44**	Noonan syndrome	*PTPN11*	Noonan syndrome—*PTPN11*
**45**	Consider other diagnosis	*-*	Noonan syndrome—*LZTR1*
**46**	Consider other diagnosis	*-*	Noonan syndrome—*RAF1*
**47**	Noonan syndrome	*PTPN11*	Noonan syndrome—*PTPN11*
**48**	Neurofibromatosis type I	*NF1*	Neurofibromatosis type I—*NF1*
**49**	Noonan syndrome	*PTPN11*	Neurofibromatosis type I—*NF1*
**50**	Neurofibromatosis type I	*NF1*	Neurofibromatosis type I—*NF1*

## Data Availability

The original contributions presented in this study are included in the article. Further inquiries can be directed to the corresponding author.

## Supplementary Materials

The following supporting information can be downloaded at https://www.mdpi.com/article/10.3390/ijms27167348/s1.
