# Supplementary material for: Proposal of an Algorithm for the Clinical and Molecular Diagnosis of RASopathies Based on HPO Nomenclature
Source: Int J Mol Sci. 2026 Aug 17;27(16):7348. doi: 10.3390/ijms27167348 (PMC13513118; doi:10.3390/ijms27167348)
Supplement: Supplementary file 1 [file ijms-27-07348-s001.zip › FINAL_04_Supplementary_Tables.pdf]

Supplementary Material

Proposal of an algorithm for the clinical and molecular diagnosis of RASopathies based on HPO nomenclature

Meneses F, Quintero C, Lores J, Gómez-Pineda E, Ramírez Montaño D, Candelo E, Pachajoa H.

Table S1. Characteristics of the validation cohort (n = 50)

All patients had a molecularly confirmed diagnosis by next-generation sequencing (NGS). Age is reported in years at the time of clinical evaluation. Algorithm Dx: diagnosis predicted by the algorithm. Misclassification reason is provided for cases where the predicted diagnosis was discordant with the molecular diagnosis. CAL: café-au-lait macules; ASD: atrial septal defect; HCM: hypertrophic cardiomyopathy; PS: pulmonic stenosis; CHD: congenital heart disease; NF1: neurofibromatosis type 1. "—" indicates no misclassification.

Patient	Dx (molecular)	Gene	Age	Sex	Algorithm Dx	Key features	Misclassification reason
P01	Noonan	PTPN11	14	M	Noonan	Pulmonic stenosis, short stature, low-set ears	—
P02	Noonan	PTPN11	7	F	Noonan	Webbed neck, ASD, ptosis	—
P03	Noonan	SOS1	5	M	Noonan	Macrocephaly, pectus excavatum, learning difficulties	—
P04	Noonan	RAF1	12	F	Other	HCM; no classic facial features	Atypical/mild phenotype
P05	Noonan	KRAS	3	M	NF1	Café-au-lait spots, no CHD	Phenotypic overlap NF1
P06	Noonan	BRAF	8	F	Noonan	Short stature, ptosis, pulmonic stenosis	—
P07	Noonan	SOS1	6	M	Noonan	Tall forehead, hypertelorism, PS	—
P08	Noonan	PTPN11	9	F	Other	Mild dysmorphic features; incomplete data	Incomplete data
P09	Noonan	RAF1	2	M	NF1	Multiple CAL macules (age-related)	Age-dependent phenotype
P10	Noonan	PTPN11	14	F	Noonan	Classic facies, ASD, short stature	—
P11	Noonan	PTPN11	11	M	Noonan	PS, triangular facies	—
P12	Noonan	KRAS	11	F	Other	No cardiac defect; borderline facial features	Atypical presentation
P13	Noonan	SOS2	5	M	Noonan	ASD, hypertelorism, short stature	—
P14	Noonan	PTPN11	7	F	Noonan	Webbed neck, PS, ptosis	—
P15	Noonan	PTPN11	13	M	Noonan	Short stature, low-set ears, ASD	—
P16	Noonan	SOS1	9	F	Noonan	Tall forehead, learning difficulties	—
P17	Noonan	PTPN11	16	M	Noonan	Classic triad: short stature, facies, cardiac	—
P18	Noonan	RAF1	4	F	Other	HCM without characteristic facial features	Atypical presentation
P19	Noonan	PTPN11	10	M	Noonan	Pectus carinatum, ASD, ptosis	—
P20	Noonan	PTPN11	12	F	Noonan	PS, triangular facies	—
P21	Noonan	SOS1	7	M	Noonan	Macrocephaly, hypertelorism, PS	—

Patient	Dx (molecular)	Gene	Age	Sex	Algorithm Dx	Key features	Misclassification reason
P22	Noonan	KRAS	5	F	NF1	Café-au-lait spots, no cardiac defect at age 5	Phenotypic overlap NF1
P23	Noonan	PTPN11	13	M	Noonan	Classic Noonan; short stature	—
P24	Noonan	PTPN11	8	F	Noonan	ASD, webbed neck, learning difficulties	—
P25	Noonan	BRAF	6	M	Noonan	Short stature, PS, ptosis	—
P26	Noonan	PTPN11	4	M	Noonan	Classic facies, PS	—
P27	Noonan	SOS1	9	F	Noonan	Tall forehead, ASD, learning difficulties	—
P28	Noonan	PTPN11	12	M	Noonan	Short stature, low-set ears, PS	—
P29	Noonan	RAF1	11	F	Other	HCM with mild facial features	Atypical/mild phenotype
P30	Noonan	PTPN11	6	M	Noonan	Webbed neck, PS	—
P31	Noonan	PTPN11	3	F	Noonan	Classic facies, ASD, short stature	—
P32	Noonan	KRAS	7	M	Noonan	Facies + PS; no CAL	—
P33	Noonan	SOS1	5	F	Noonan	Macrocephaly, learning difficulties, ASD	—
P34	Noonan	PTPN11	10	M	Noonan	Classic Noonan; short stature, webbed neck	—
P35	Noonan	PTPN11	8	F	Noonan	Pectus, PS, ptosis	—
P36	NF1	NF1	6	M	NF1	≥6 CAL, Lisch nodules, freckling	—
P37	NF1	NF1	9	F	NF1	CAL, neurofibromas, freckling	—
P38	NF1	NF1	4	M	NF1	CAL, Lisch nodules	—
P39	NF1	NF1	12	F	NF1	Plexiform neurofibroma, CAL, freckling	—
P40	NF1	NF1	7	M	NF1	CAL, axillary freckling	—
P41	NF1	NF1	3	F	Noonan	CAL absent at age 3; short stature, low-set ears	Age-dependent; early NF1
P42	NF1	NF1	15	M	NF1	Multiple neurofibromas, Lisch nodules	—
P43	NF1	NF1	8	F	NF1	CAL, freckling, optic glioma	—
P44	NF1	NF1	5	M	NF1	CAL, macrocephaly, Lisch nodules	—
P45	NF1	NF1	11	F	NF1	Plexiform neurofibroma, CAL	—
P46	NF1	NF1	2	M	NF1	CAL; Lisch not yet visible	—
P47	NF1	NF1	6	F	NF1	≥6 CAL, freckling	—
P48	NF1	NF1	10	M	NF1	Neurofibroma, CAL, optic glioma	—
P49	NF1	NF1	4	F	NF1	CAL, axillary freckling	—
P50	NF1	NF1	9	M	NF1	CAL, Lisch nodules, macrocephaly	—

Note: Overall algorithm accuracy: 78% (39/50; 95% CI 64.0–88.4%) for clinical syndrome prediction and 66% (33/50; 95% CI 51.2–78.8%) for molecular gene prediction. Confidence intervals calculated using the Wilson score method.

Table S2. Inter-rater agreement between the two researchers (n = 50)

Both researchers independently applied the algorithm to blinded phenotypic data for each of the 50 patients. Discrepancies were subsequently identified and resolved by consensus. Inter-rater agreement was assessed using Cohen's kappa coefficient (κ = 0.82; 95% CI: 0.71–0.93), indicating strong agreement. ✓: agreement. ✗: initial disagreement (resolved by consensus; final consensus classification used for all accuracy calculations).

Patient	Researcher 1	Researcher 2	Agreement
P01	Noonan	Noonan	✓
P02	Noonan	Noonan	✓
P03	Noonan	Noonan	✓
P04	Other	Other	✓
P05	NF1	Other	✗
P06	Noonan	Noonan	✓
P07	Noonan	Noonan	✓
P08	Other	Other	✓
P09	NF1	Other	✗
P10	Noonan	Noonan	✓
P11	Noonan	Noonan	✓
P12	Other	Other	✓
P13	Noonan	Noonan	✓
P14	Noonan	Noonan	✓
P15	Noonan	Noonan	✓
P16	Noonan	Noonan	✓
P17	Noonan	Noonan	✓
P18	Other	Other	✓
P19	Noonan	Noonan	✓
P20	Noonan	Noonan	✓
P21	Noonan	Noonan	✓
P22	NF1	Other	✗
P23	Noonan	Noonan	✓
P24	Noonan	Noonan	✓
P25	Noonan	Noonan	✓
P26	Noonan	Noonan	✓
P27	Noonan	Noonan	✓
P28	Noonan	Other	✗

Patient	Researcher 1	Researcher 2	Agreement
P29	Other	Other	✓
P30	Noonan	Noonan	✓
P31	Noonan	Noonan	✓
P32	Noonan	Noonan	✓
P33	Noonan	Noonan	✓
P34	Noonan	Noonan	✓
P35	Noonan	Noonan	✓
P36	NF1	NF1	✓
P37	NF1	NF1	✓
P38	NF1	NF1	✓
P39	NF1	NF1	✓
P40	NF1	NF1	✓
P41	Noonan	Other	✗
P42	NF1	NF1	✓
P43	NF1	NF1	✓
P44	NF1	NF1	✓
P45	NF1	NF1	✓
P46	NF1	NF1	✓
P47	NF1	NF1	✓
P48	NF1	NF1	✓
P49	NF1	NF1	✓
P50	NF1	NF1	✓

Note: Five discrepancies were observed across 50 cases (overall percent agreement: 90.0%). All discordant cases were resolved by consensus prior to final classification.

Table S3. Multiclass confusion matrix for algorithm predictions

The confusion matrix summarises the concordance between algorithm-predicted diagnoses and molecularly confirmed diagnoses across all 50 patients. Because the validation cohort comprised exclusively molecularly confirmed RASopathy cases, no true-negative individuals were available; specificity and negative predictive value therefore cannot be estimated.

Molecular Dx	Predicted: Noonan	Predicted: NF1	Predicted: Other
Noonan (n = 35)	24 (68.6%)	3 (8.6%)	8 (22.9%)
NF1 (n = 15)	1 (6.7%)	13 (86.7%)	1 (6.7%)

Performance metrics by syndrome:

• Noonan syndrome: sensitivity 68.6%, positive predictive value (PPV) 96.0%, F1-score 0.80

• Neurofibromatosis type 1: sensitivity 86.7%, PPV 81.3%, F1-score 0.84

• Overall accuracy: 78% (95% CI: 64.0–88.4%; Wilson score method)

Table S4. GeneReviews sources used for phenotype frequency extraction

For each RASopathy, the "Frequency of Select Features" section of the corresponding GeneReviews chapter was used as the primary reference source for phenotype frequency data. The HPO database was accessed in January 2024. All chapters were verified active as of July 2026.

RASopathy	Causative genes	GeneReviews chapter	NCBI Bookshelf ID
Noonan syndrome	PTPN11, SOS1, RAF1, KRAS, BRAF, NRAS, RIT1, SOS2, LZTR1, MRAS	van der Burgt I, et al.	NBK1124
Cardiofaciocutaneous syndrome	BRAF, MAP2K1, MAP2K2, KRAS	Roberts AE, Allanson JE, et al.	NBK1186
Legius syndrome	SPRED1	Brems H, et al.	NBK47312
Noonan with Multiple Lentigines	PTPN11, RAF1	Sarkozy A, et al.	NBK1383
Costello syndrome	HRAS	Gripp KW, Lin AE, et al.	NBK1507
Neurofibromatosis type 1	NF1	Ferner RE, et al.	NBK1109

Note: All chapters accessible via NCBI Bookshelf (https://www.ncbi.nlm.nih.gov/books/).

Supplementary References

Rath A, Salamon V, Peixoto S, et al. A systematic literature review of evidence-based clinical practice for rare diseases: what are the perceived and real barriers for improving the evidence and how can they be overcome? Trials. 2017;18(1):556. doi:10.1186/s13063-017-2287-7

Partington G, Cro S, Mason A, et al. Design and analysis features used in small population and rare disease trials: A targeted review. J Clin Epidemiol. 2022;144:93–101. doi:10.1016/j.jclinepi.2021.12.009
