# Supplementary material for: Proposal of an Algorithm for the Clinical and Molecular Diagnosis of RASopathies Based on HPO Nomenclature
Source: Int J Mol Sci. 2026 Aug 17;27(16):7348. doi: 10.3390/ijms27167348 (PMC13513118; doi:10.3390/ijms27167348)
Supplement: Supplementary file 1 [file ijms-27-07348-s001.zip › FINAL_03_Supplementary_Figures.pdf]

Supplementary Material

Proposal of an algorithm for the clinical and molecular diagnosis of RASopathies based on HPO nomenclature

Meneses F, Quintero C, Lores J, Gómez-Pineda E, Ramírez Montaño D, Candelo E, Pachajoa H.

Figure S1. RASopathy Diagnostic Algorithm

The algorithm provides a stepwise, HPO-based decision-tree framework for the clinical and molecular evaluation of patients with suspected RASopathies, beginning with the assessment of short stature. For each syndrome, compatible clinical findings are evaluated sequentially; a positive finding directs the clinician toward the corresponding clinical and molecular diagnosis, while a negative finding advances to the next syndrome in the hierarchy. Patients who are negative for all six syndromes are directed toward consideration of alternative diagnoses.

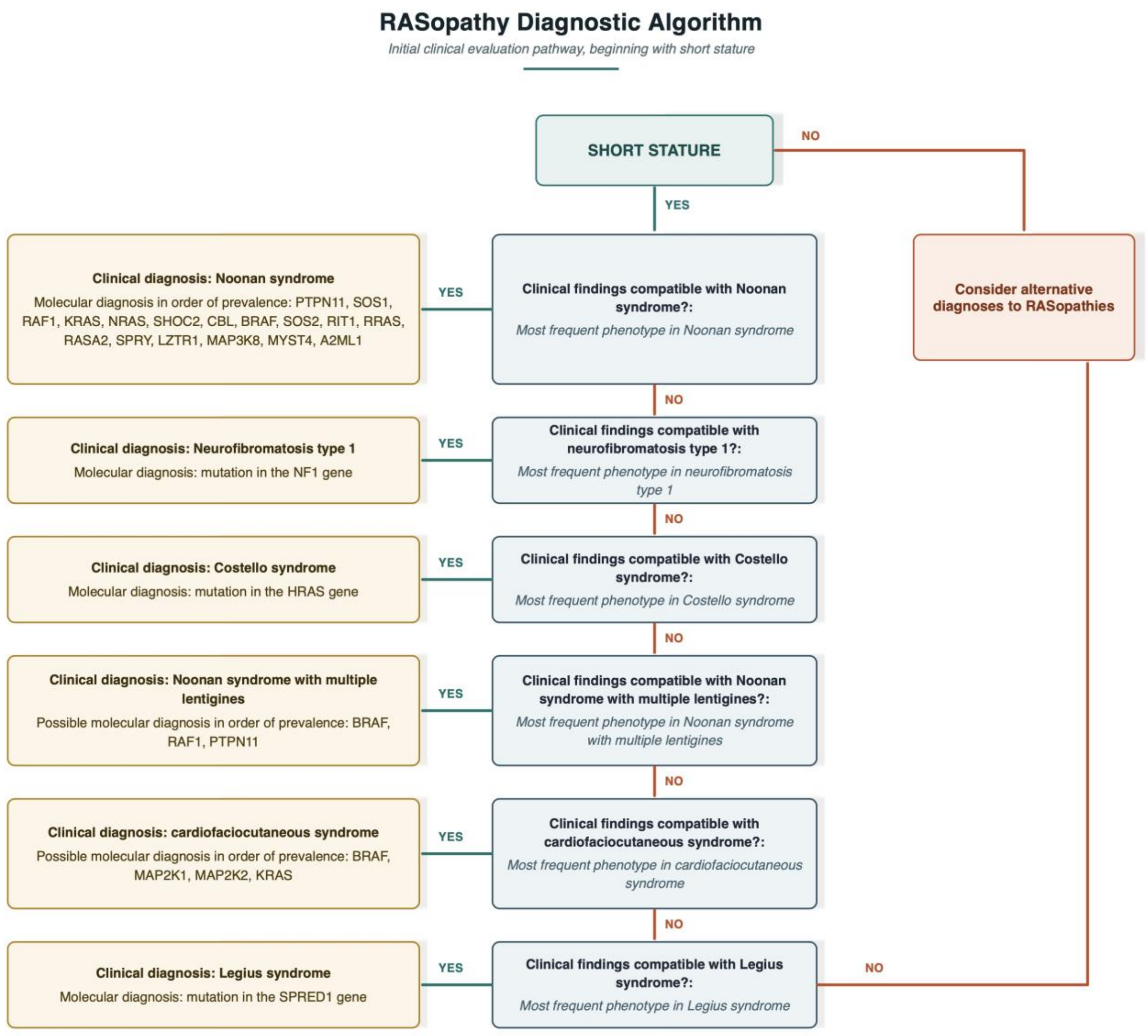

Figure S1. Stepwise diagnostic decision tree beginning with short stature as the initial clinical criterion. Clinical findings are evaluated sequentially for six RASopathy subtypes: Noonan syndrome (genes: PTPN11, SOS1, RAF1, KRAS, NRAS, SHOC2, CBL, BRAF, SOS2, RIT1, RRAS, RASA2, SPRY, LZTR1, MAP3K8, MYST4, A2ML1), neurofibromatosis type 1 (NF1), Costello syndrome (HRAS), Noonan syndrome with multiple lentigines (BRAF, RAF1, PTPN11), cardiofaciocutaneous syndrome (BRAF, MAP2K1, MAP2K2, KRAS), and Legius syndrome (SPRED1). Patients who are negative for all criteria are directed to consider alternative diagnoses. Phenotype frequencies are based on the HPO database (accessed January 2024) and GeneReviews.

Figure S2. Step-by-step clinical use pathway for the RASopathy diagnostic algorithm

The pathway illustrates the proposed workflow from initial patient presentation to molecular confirmation, genetic counselling, and syndrome-specific clinical management. The algorithm (Step 3) is applied by the clinician following initial phenotypic assessment using Human Phenotype Ontology (HPO) terminology. A high-probability syndrome assignment guides referral to a clinical geneticist (Step 4) and subsequent molecular confirmation by next-generation sequencing (Step 5). Confirmed cases receive genetic counselling (Step 6) and syndrome-specific clinical management (Step 7).

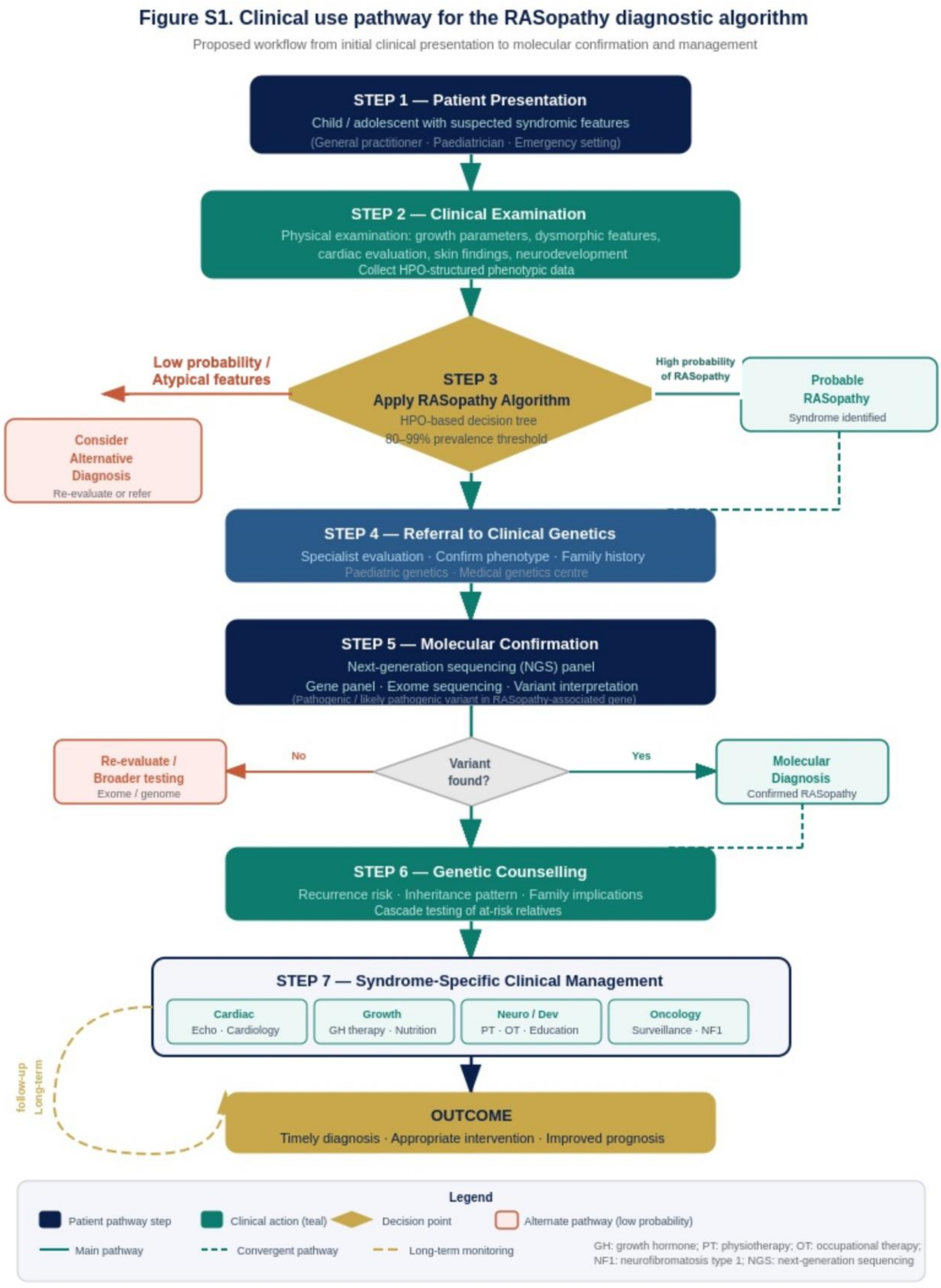

Figure S2. Clinical use pathway for the RASopathy diagnostic algorithm. Steps are numbered 1–7 from initial patient presentation (Step 1) to long-term follow-up (Step 7). Decision points (diamond shapes) represent algorithm output or molecular result. Teal boxes indicate clinical actions; dark navy boxes indicate key diagnostic milestones. Red-outlined boxes indicate low-probability pathways requiring alternative evaluation. The cyclic gold dashed arrow represents long-term clinical monitoring. GH: growth hormone; HPO: Human Phenotype Ontology; NF1: neurofibromatosis type 1; NGS: next-generation sequencing; OT: occupational therapy; PT: physiotherapy.
